# Electrochemical Characterization of a Molecularly Imprinted Polymer Sensor for the Selective Recognition of Type II Collagen in Joint Degeneration Monitoring

**DOI:** 10.3390/polym18030321

**Published:** 2026-01-25

**Authors:** Jindapa Nampeng, Naphatsawan Vongmanee, Chuchart Pintavirooj, Sarinporn Visitsattapongse

**Affiliations:** Department of Biomedical Engineering, School of Engineering, King Mongkut’s Institute of Technology Ladkrabang, Bangkok 10520, Thailand; jindapa.na@kmitl.ac.th (J.N.); naphatsawan.v@hotmail.com (N.V.); chuchart.pi@kmitl.ac.th (C.P.)

**Keywords:** molecularly imprinted polymer, electrochemical biosensor, screen-printed electrode, arthritis biomarkers, collagen type II, cartilage degradation, joint degeneration monitoring, polymerization

## Abstract

Type II collagen is a primary fibrillar component of articular cartilage, and its early degradation is a key biomarker of joint-degenerative disorders such as osteoarthritis, rheumatoid arthritis, gout, etc. Reliable detection at low concentrations remains challenging due to limited assay accessibility, complex analytical procedures, and nonspecific responses in multicomponent biological matrices. This research reports the development of a Molecularly Imprinted Polymer (MIP)–based electrochemical sensor engineered for the selective recognition of type II collagen. A series of monomer formulations were evaluated, and the 1AAM:2VP composition produced a well-defined imprinted layer on screen-printed carbon electrodes, yielding the highest electrochemical sensitivity and linearity. The optimized sensor exhibited strong anodic and cathodic responses proportional to increasing collagen concentrations, with a calibration slope corresponding to an R^2^ value of 0.9394. Minimal signal interference was observed, confirming high molecular selectivity. The limit of detection (LOD) was calculated to be approximately 0.065 µg/mL. These characteristics demonstrate that the proposed MIP sensor provides a low-cost, accessible, and highly selective analytical platform suitable for early-stage cartilage degeneration monitoring.

## 1. Introduction

Type II collagen is the most abundant structural protein within human articular cartilage [1], where it provides mechanical strength and helps maintain tissue shape, resilience, and load-bearing capacity necessary for smooth, pain-free joint movement. During the early stages of cartilage degradation, fragments of type II collagen are released into synovial fluid and, in more advanced cases, into the bloodstream. The presence of these fragments represents one of the earliest measurable biochemical indicators of degenerative joint diseases (DJD), a group of musculoskeletal disorders characterized by progressive cartilage degradation, including osteoarthritis (OA) [2,3], rheumatoid arthritis (RA) [1], and related inflammatory and degenerative joint conditions, providing direct evidence of cartilage matrix breakdown well before macroscopic or radiographic changes become clinically apparent. Owing to this strong association, type II collagen has been widely recognized as a key biomarker for assessing cartilage degeneration and disease progression. The development and progression of DJD are influenced by multiple factors, including genetic susceptibility, previous joint injury, chronic inflammatory processes, aging, sex-related differences, and obesity [4]. As type II collagen degrades, the cartilage matrix progressively weakens, ultimately resulting in pain, stiffness, and reduced joint mobility. Consequently, monitoring biochemical changes associated with type II collagen plays a crucial role in detecting early cartilage degeneration and supporting timely clinical or therapeutic decision-making.

Despite ongoing advances, current diagnostic tools remain limited in their ability to detect early-stage cartilage damage. Commonly used methods—such as enzyme-linked immunosorbent assays (ELISA), T2CM assays for MMP-derived type II collagen fragments, polymerase chain reaction (PCR), ultraperformance liquid chromatography–tandem mass spectrometry (UPLC-MS/MS), and temporomandibular joint (TMJ) panoramic radiography—often struggle to identify biochemical cartilage degradation at an early stage. Many of these approaches are designed for specific DJD subtypes, offer limited accessibility, or rely on complex, time-consuming, and laboratory-dependent procedures. In addition, these methods are frequently costly and tend to detect disease only after substantial structural damage has already occurred, thereby limiting opportunities for early intervention. Recent studies have demonstrated the growing potential of molecularly imprinted polymer (MIP) technology for early biomarker detection in biomedical diagnostics, particularly due to its robustness, stability, and cost efficiency [2,3]. However, most reported MIP-based sensors focus on cancer or small-molecule biomarkers and generally lack sufficient selectivity toward type II collagen, the principal molecular component associated with cartilage degeneration in DJD [1,2,5]. These limitations highlight the need for diagnostic tools that are more affordable, highly selective, accessible, and capable of detecting early biochemical degradation to slow disease progression and improve long-term patient outcomes.

In response to these challenges, this study presents an MIP-based electrochemical sensor designed for the selective recognition of type II collagen in the context of joint degeneration monitoring. The underlying concept exploits the ability of MIP technology to create artificial binding cavities within a polymer matrix that are complementary in size, shape, and functional group orientation to the target molecule, mimicking natural recognition mechanisms such as antigen–antibody interactions [1,2,5,6,7,8,9]. Extensive literature has demonstrated that MIP-based biosensors provide significant advantages over conventional biological receptors, including enhanced chemical stability, reproducibility, resistance to harsh environments, and long shelf life, making them particularly attractive for point-of-care and real-world diagnostic applications [3]. In addition, MIP-based electrochemical sensors have shown strong potential for early-stage detection with high sensitivity and selectivity across diverse biomedical targets [2]. In this study, a carbon screen-printed electrode (SPE) was selected as the sensing substrate due to its favourable electrochemical performance, scalability, and compatibility with polymer-based surface modification. The incorporation of graphene oxide (GO)-enhanced carbon surfaces further improves signal quality, charge transfer efficiency, stability, and cost-effectiveness, as widely reported in recent biosensor literature. The polymer layer was fabricated using two functional monomers, N-vinylpyrrolidone (VP) and acrylamide (AAM), with dimethyl sulfoxide (DMSO) as the solvent, ethylene glycol dimethacrylate (EGDMA) as the crosslinker, and azobisisobutyronitrile (AIBN) as the free-radical initiator. Purified bovine type II collagen was employed as the template molecule to generate complementary binding sites within the polymer matrix. UV curing and controlled thermal incubation were applied to strengthen and stabilize the polymer network on the SPE surface, followed by template removal using 5% (*w*/*v*) acetic acid to expose specific recognition cavities corresponding to the target molecule. Electrochemical behaviour was evaluated using cyclic voltammetry with a Metrohm DropSens system (DRP-220BT, Asturias, Spain) and DropView 8400 software, monitoring changes in anodic and cathodic current in relation to collagen concentration [2,3,4,5,6,10].

This study aims to address the limitations of existing diagnostic methods by optimizing a high-performance MIP-based electrochemical sensor capable of selectively recognizing type II collagen with high sensitivity for DJD assessment. The sensor was evaluated across a concentration range of 0.01–100 µg/mL, achieving a limit of detection (LOD) of 0.065 µg/mL, demonstrating strong sensitivity at low analyte concentrations relevant to early-stage cartilage degradation. Furthermore, specificity analyses confirmed minimal response toward non-target collagens, validating the selective binding behaviours of the imprinted polymer network. Overall, these findings support the potential of this MIP-based electrochemical platform as a more accessible, highly selective, and early-stage diagnostic tool for monitoring type II collagen degradation in degenerative joint conditions.

## 2. Materials and Methods

### 2.1. Collagen Peptides

Collagen-based molecular imprinting was designed using fibrillar collagens with closely related structures but distinct physiological roles to evaluate selective molecular recognition. Type II collagen (Sigma-Aldrich, St. Louis, MO, USA) was chosen as the target analyte due to its predominance in articular cartilage and its relevance as a biomarker of cartilage degradation [8]. Type I and type III collagens (Sigma-Aldrich, USA) were employed as structurally related non-target controls to assess sensor specificity, given their overlapping triple-helical architecture and partial co-distribution in connective tissues [8,10].

**Figure 1:**
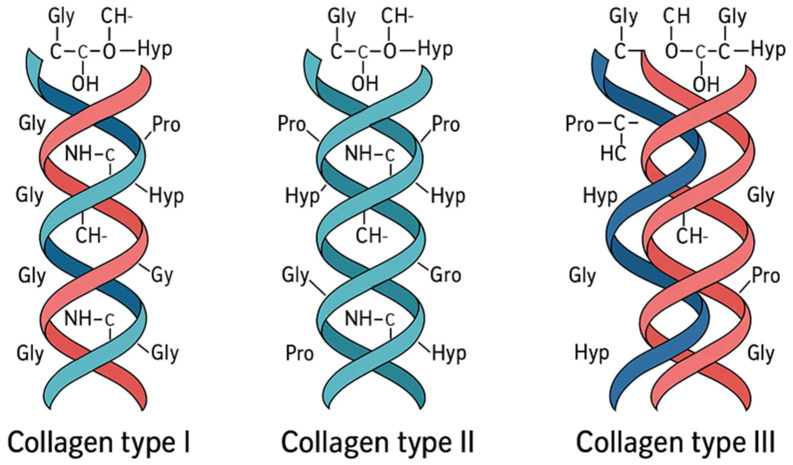
Illustrates the molecular configuration of collagen types I (**left**), II (**centre**), and III (**right**), highlighting their triple-helix arrangement and compositional variations that influence their biochemical and mechanical properties.

In this study, type I, type II, and type III collagens were selected due to their overlapping biological distribution and structural resemblance as shown in Figure 1, yet with distinct physiological roles. Type I (Sigma-Aldrich, USA) predominates in skin, bone, and tendon while type II is the major component of articular cartilage and intervertebral discs, whereas type III is abundant in blood vessels, skin, and soft connective tissues, often co-fibrillizing with type I. These subtle differences in amino-acid composition and fibrillar organization are critical for selective molecular recognition in biosensor applications.

In this study, purified bovine type II collagen was used as the template molecule because of its high structural and biochemical homology to human type II collagen, enabling reproducible imprinting while avoiding ethical and regulatory constraints associated with human-derived materials. For specificity analysis, bovine type I collagen and a commercially available type I & III collagen blend were utilized, as pure type III collagen is rarely available due to its natural co-fibrillization with type I and its limited structural stability when isolated.

The molecularly imprinted polymer (MIP) layer was synthesized by free-radical polymerization using type II collagen as the template molecule. Acrylamide (AAM) and N-vinylpyrrolidone (VP) were employed as functional monomers at a molar ratio of 1:2. Non-covalent interactions between the collagen template and functional monomers, including hydrogen bonding and dipole–dipole interactions, enabled the formation of a stable pre-polymerization complex. Dimethyl sulfoxide (DMSO) was used as the solvent to dissolve the collagen template and polymerization components, ensuring homogeneous dispersion and favorable monomer-template interactions. Ethylene glycol dimethacrylate (EGDMA) was used as the crosslinking agent to stabilize the three-dimensional polymer network, while azobisisobutyronitrile (AIBN) served as the thermal radical initiator.

Type II collagen was dissolved in DMSO and stirred for 8–12 h to obtain a homogeneous solution. The functional monomers, crosslinker, and initiator were then added sequentially to prepare the pre-polymerization mixture. Polymerization was initiated by heating the solution at 37 °C for 45 min, during which AIBN-generated radicals promoted chain propagation and crosslinking, resulting in a crosslinked polymer matrix embedding the collagen template. Template removal was subsequently performed as described in the fabrication procedure to generate collagen-specific imprinted cavities.

### 2.2. Screen Printed Electrode Fabrication

**Figure 2:**
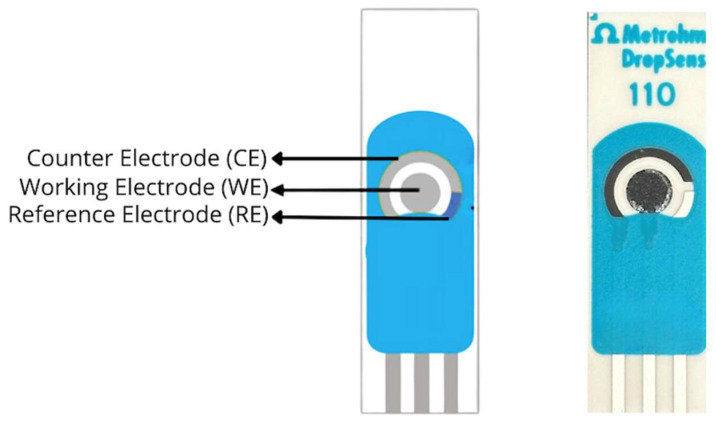
Schematic and image of a three-electrode screen-printed electrode area (WE (Grey), CE (Grey), and RE (Blue) used for electrochemical measurements.

A carbon screen-printed electrode (SPE) was selected as the sensing platform regarding to its compact design, low cost, and suitability for electrochemical polymerization. The electrode consists of a three-electrode configuration including a working electrode, a reference electrode, and a counter electrode, integrated onto a single substrate to ensure precise potential control and reproducible signal acquisition as illustrated in Figure 2. This configuration enables stable signal measurement with minimal background interference [11,12,13]. The working electrode plays as the active sensing area, where the MIP layer is deposited. The polymer layer is synthesized to contain specific recognition sites corresponding to type II collagen, which acts as the template molecule during the imprinting process. After polymerization, the collagen template is removed to expose the imprinted cavities. These cavities possess both shape-like and chemical complementarity to the target molecule, ensuring selective rebinding of type II collagen during measurement.

**Figure 3:**
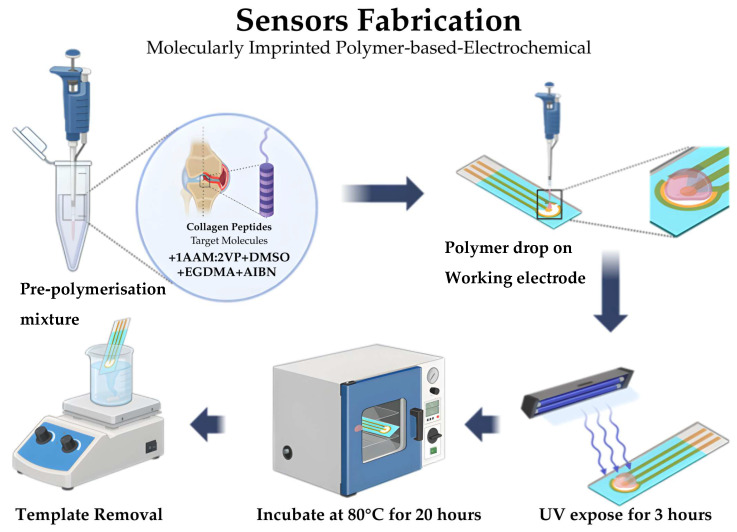
Schematic illustration of the fabrication process for the molecularly imprinted polymer–based electrochemical sensor on a carbon screen-printed electrode.

Figure 3 summarizes the fabrication procedure of the molecularly imprinted polymer (MIP) biosensor on a carbon screen-printed electrode (SPE). Carbon SPEs were selected due to their low cost, disposability, and compatibility with electrochemical sensing. Each SPE consisted of an integrated three-electrode system comprising a carbon working electrode, a carbon counter electrode, and a silver/silver chloride reference electrode printed on a single planar substrate.

The MIP pre-polymer solution was prepared by dissolving collagen peptides (template molecule) in dimethyl sulfoxide (DMSO), followed by the addition of acrylamide (AAM) and 2-vinylpyridine (VP) at a molar ratio of 1:2. Ethylene glycol dimethacrylate (EGDMA) was used as the crosslinker, and azobisisobutyronitrile (AIBN) served as the radical initiator. The resulting homogeneous solution was drop-cast onto the carbon working electrode surface (approximately 5–10 µL) and allowed to spread naturally to form a uniform polymer layer. Polymerization and film stabilization were achieved through sequential curing steps. The coated electrodes were exposed to ultraviolet (UV) irradiation for 3 h, followed by thermal incubation at 80 °C for 18–20 h to ensure complete polymer consolidation and solvent removal. After curing, the collagen template was removed by immersing the modified electrodes in 5% (*v*/*v*) acetic acid for 30 min, followed by thorough rinsing with distilled water. This process generated selective imprinted cavities within the polymer matrix while preserving film integrity. Electrochemical measurements were performed using a reversible redox probe solution consisting of 5.0 mM potassium ferricyanide [K_3_Fe(CN)_6_] prepared in 0.1 M phosphate-buffered saline (PBS, pH 7.4). The buffer solution was used to maintain constant ionic strength and physiological pH during measurements. All electrochemical analyses were conducted at room temperature using freshly prepared solutions. Prior to each measurement, 100 µL of the redox probe solution was drop-cast onto the electrode surface, ensuring full coverage of the working electrode area.

### 2.3. Electrochemical Signal Measurement

Electrochemical characterization of the fabricated MIP-based sensor was performed using cyclic voltammetry (CV) to evaluate the redox behavior of the system before and after the binding of type II collagen. CV provides information on electron-transfer kinetics and interfacial changes occurring at the modified electrode surface. During each potential sweep, the working electrode is subjected to a cyclic variation in voltage while the corresponding current response is recorded, producing a voltammogram that reflects the oxidation–reduction processes involved.

**Figure 4:**
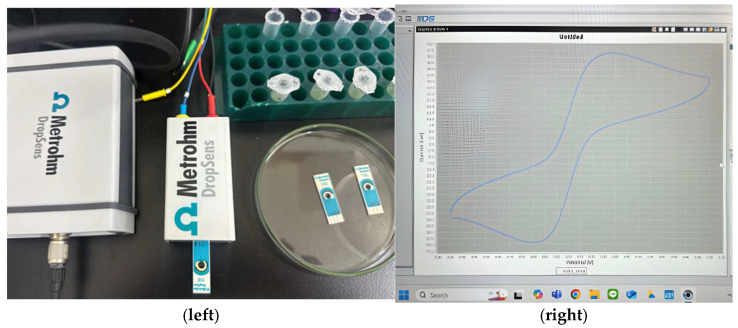
Electrode being measured in the experimental setup (**left**) and the corresponding cyclic voltammetry electrochemical signal (**right**).

Electrochemical measurements were performed using DropView 8400 software connected to the electrochemical workstation as shown in Figure 4. The modified carbon screen-printed electrode served as the working electrode, with the integrated reference and counter electrodes enabling stable and reproducible measurements. The potential was scanned from −0.5 V to +0.7 V at a scan rate of 0.05 V/s. These parameters were selected to ensure adequate coverage of the redox-active potential window while minimizing background noise and capacitive effects. All cyclic voltammograms were recorded under identical experimental conditions to allow direct comparison between different stages of sensor fabrication, including the bare electrode, MIP-modified electrode, template-removed surface, and target-bound state.

The recorded voltammograms typically display two characteristic peaks: an anodic peak and a cathodic peak. The anodic peak corresponds to the oxidation process, during which electrons are transferred from the redox-active species to the electrode surface. Conversely, the cathodic peak represents the reduction process, where electrons are transferred back from the electrode to the redox species. The presence of well-defined and symmetric anodic and cathodic peaks indicates reversible or quasi-reversible electron-transfer behavior. The magnitude of the peak currents and the separation between anodic and cathodic peaks serve as important indicators of electron-transfer kinetics and surface conductivity. A higher peak current generally reflects improved charge-transfer efficiency and easier access of redox species to the electrode surface, whereas reduced peak current suggests restricted diffusion or increased interfacial resistance. Following deposition of the molecularly imprinted polymer layer, a noticeable decrease in both anodic and cathodic peak currents was observed compared with the bare electrode. This reduction in current is attributed to the formation of a polymer layer that partially blocks electron transfer and limits diffusion of redox species to the electrode surface. The insulating nature of the polymer matrix introduces an additional barrier to charge transfer, confirming successful modification of the electrode surface. After removal of the collagen template, partial recovery of the peak current was typically observed. This recovery indicates the formation of recognition cavities within the polymer matrix, which reopen diffusion pathways and allow redox species to approach the electrode surface more easily. The difference in current response before and after template removal provides indirect evidence that the imprinting process successfully generated accessible cavities. When type II collagen was introduced to the MIP-modified electrode, further changes in the cyclic voltammograms were observed. Specific binding of collagen molecules within the imprinted cavities alters the local electron environment and restricts diffusion of redox species near the electrode interface. As a result, a decrease in peak current intensity was detected, reflecting hindered charge transfer caused by target rebinding. This reduction in current becomes more pronounced with increasing concentrations of type II collagen, demonstrating a concentration-dependent electrochemical response. The correlation between peak current variation and target concentration confirms that the observed signal changes arise from specific molecular recognition rather than nonspecific adsorption. In contrast, non-imprinted polymer (NIP) electrodes typically exhibit smaller or negligible signal changes under identical conditions, further supporting the selectivity of the MIP sensor.

Repeated cyclic voltammetry scans were conducted to assess the reproducibility and stability of the fabricated sensor. The consistent shape and position of anodic and cathodic peaks across multiple cycles indicate stable electrochemical behavior and strong adhesion of the polymer layer to the electrode surface. Minimal signal drift was observed, confirming that the MIP layer remained intact during electrochemical interrogation. This reproducible peak response is essential for quantitative sensing applications, as it ensures reliable signal interpretation and minimizes measurement uncertainty. The stability observed during CV measurements demonstrates that the fabricated sensor is suitable for repeated analysis and long-term monitoring applications.

Cyclic voltammetry plays a crucial role in evaluating the performance of MIP-based electrochemical sensors. It provides rapid and intuitive insight into surface modification, cavity formation, and target binding behavior. By monitoring changes in peak current and peak separation, CV allows assessment of polymer conductivity, recognition efficiency, and interfacial electron-transfer properties. Although CV is often considered a qualitative or semi-quantitative technique, it provides essential foundational information that supports more sensitive analytical methods such as differential pulse voltammetry or electrochemical impedance spectroscopy. When used in combination with these techniques, CV contributes to a comprehensive understanding of sensor behavior. In summary, cyclic voltammetry was successfully employed to characterize the electrochemical behavior of the MIP-based sensor and to verify selective binding of type II collagen. The observed changes in anodic and cathodic peak currents following polymer formation, template removal, and target rebinding confirm effective imprinting and functional recognition. The consistent and reproducible electrochemical response highlights the stability and reliability of the fabricated sensor, supporting its potential application in quantitative monitoring of collagen-related joint degeneration.

## 3. Results

### 3.1. Sensors Optimization and Characteristic

In this study, several functional monomers were evaluated for their suitability in the molecular imprinting process, including Acrylamide (AAM), Methacrylic acid (MAA), 4-Vinylpyridine (4-VP), and Hydroxyethyl methacrylate (HEMA). Each monomer was examined in varying ratios to assess its interaction efficiency with the collagen template and its effect on polymer structure. Among these, Acrylamide (AAM) and 4-Vinylpyridine (4-VP) demonstrated the most favorable physicochemical compatibility, providing strong hydrogen bonding, electrostatic interaction, and chemical stability during the imprinting process. In contrast, MAA, though widely used in MIP systems exhibits excessive acidity that can lead to nonspecific ionic interactions and partial denaturation of protein templates such as collagen. HEMA, while hydrophilic, provides weaker binding energy and limited hydrogen-bond donor sites, resulting in lower imprint stability and reduced electrochemical sensitivity. The combined use of AAM and 4-VP yielded a more selective, stable, and conductive polymer network, leading to improved signal amplification and reproducible recognition behavior in the electrochemical sensor. These characteristics led to their selection as the primary functional monomers for further optimization.

The recognition of type II collagen by the molecularly imprinted polymer (MIP) relies on both chemical affinity and structural matching that are formed during the imprinting process and reused during sensing. During MIP preparation, type II collagen serves as the template and interacts with functional monomers such as AAM and 4-VP through a free-radical copolymerization reaction as noncovalent forces, including hydrogen bonding, electrostatic attraction, and polar interactions. Functional groups on type II collagen, such as amide (–CONH–), carboxyl (–COOH), and hydroxyl (–OH) groups, temporarily interact with complementary sites on the monomers, allowing the polymer network to organize around the template in a favorable arrangement before polymerization. As polymerization proceeds, the growing AAM-4-VP copolymer chains become crosslinked into a stable three-dimensional network. After polymerization and removal of the template, well-defined recognition cavities remain within the polymer matrix. These cavities match type II collagen in terms of size, shape, and the spatial distribution of functional groups. When type II collagen is reintroduced, it selectively fits into these cavities and reforms the same interactions created during imprinting, enabling discrimination from other proteins or non-target collagen types. From an electrochemical perspective, the rebinding of type II collagen to the MIP layer partially blocks the electrode surface and hinders the diffusion of the redox probe. This leads to a decrease in the measured current, with the magnitude of the signal change directly reflecting the strength and efficiency of the binding between type II collagen and the imprinted sites.

To evaluate the electrochemical performance of the fabricated MIP-based biosensors, three optimized polymer conditions were prepared and analyzed using different monomer ratios: Condition 1 (1AAM:1VP), Condition 2 (1AAM:2VP), and Condition 3 (2AAM:1VP) as shown in Table 1. The electrochemical responses were examined through cyclic voltammetry (CV), focusing on variations in current intensity in response to increasing concentrations of type II collagen, ranging from 0.01 to 100 µg/mL. For each concentration level, measurements were conducted in quintuplicate (*n* = 5) to ensure statistical reliability and reproducibility. Each composition exhibited a unique electrochemical profile, demonstrating the impact of monomer proportion on polymer–template recognition, charge transfer behavior, and binding site stability.

In Condition 1 (1AAM:1VP), the voltametric signal showed a proportional increase in current with rising collagen concentration, indicating successful recognition by the MIP layer. However, the overall percentage change remained moderate, with a maximum current shift of approximately 28% at the highest tested concentration. The signal exhibited stable but relatively low sensitivity across the measured range, suggesting a limited density of active recognition sites and suboptimal hydrogen bonding between the monomers and the collagen template.

For Condition 3 (2AAM:1VP), a similar increasing trend was observed, though with slightly higher current responses compared to Condition 1. The signal reached nearly 30% at higher concentrations, indicating improved interaction due to the increased proportion of AAM, which provides a hydrogel-like microenvironment favorable for protein binding. However, at lower concentrations, the signal consistency decreased, implying weaker site uniformity and reduced reproducibility. This behavior may be attributed to excessive AAM content, which can lead to denser polymer networks and hinder effective mass transfer within the sensing layer.

The most significant improvement was achieved with Condition 2 (1AAM:2VP), which demonstrated a consistent and substantial signal increase across all collagen concentrations. The relative current change reached 34.7% at 100 μg/mL, representing the highest response among the tested formulations. This composition achieved a balance between flexibility (from AAM) and biocompatibility (from VP), resulting in a well-structured polymer network with enhanced template accessibility. The increased proportion of 4-VP improved π–π and dipole–dipole interactions with the amide and hydroxyl groups of collagens, supporting stronger electron transfer and more stable template rebinding. This balance between AAM’s flexibility and VP’s electroactivity created a highly conductive and selective MIP layer, confirming Condition 2 as the optimal formulation for reliable detection of type II collagen.

**Figure 5:**
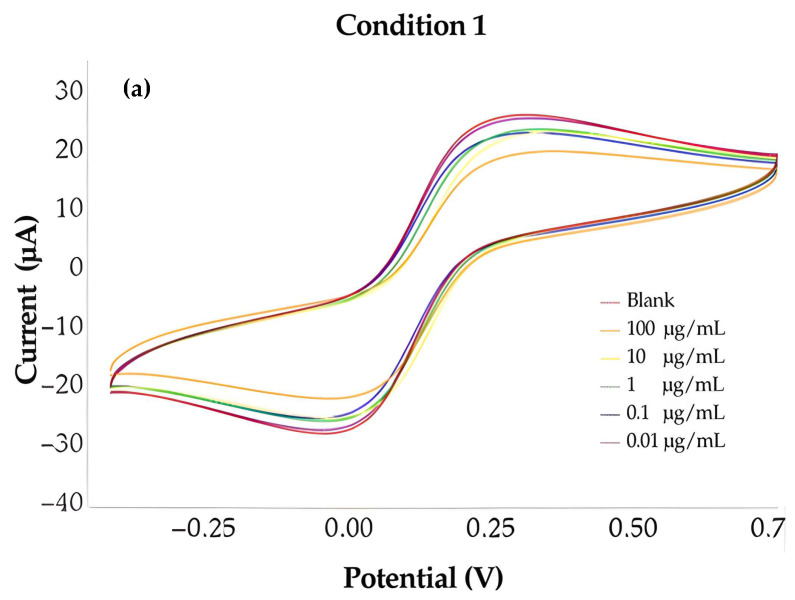

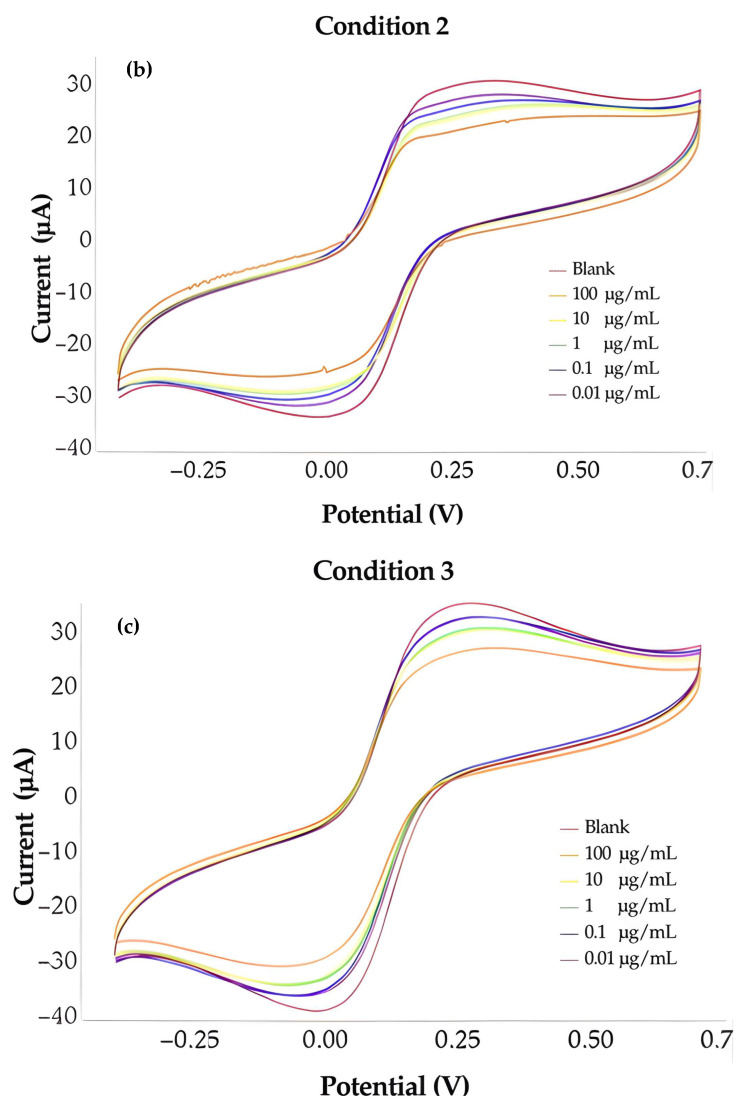
Cyclic voltammetry (CV) responses of the MIP-modified electrode recorded under (**a**) Condition 1, (**b**) Condition 2, and (**c**) Condition 3 across varying collagen concentrations.

In Condition 1 (1AAM:1VP), the cyclic voltammetry profiles display only modest variations as collagen concentration increases. The anodic and cathodic features remain closely aligned, with minimal peak displacement and relatively narrow separation between the forward and reverse scans as shown in Figure 5a. Such behavior suggests that the polymer network formed under this monomer ratio provides limited modulation of electron-transfer dynamics upon collagen binding. The restrained peak evolution indicates a lower density of effective recognition sites and weaker template; polymer interactions compared with the other formulations. Consequently, Condition 1 demonstrates moderate sensitivity, reflecting a less responsive interfacial environment and reduced amplification of current changes across the tested concentration range.

In Condition 2 (1AAM:2VP), the cyclic voltammetry profiles reveal a markedly enhanced electrochemical response. As the collagen concentration increases, the CV curves display a clear and progressive shift in both anodic and cathodic peak intensities, along with widening of the peak-to-peak separation that illustrate in Figure 5b. This pronounced evolution reflects a more efficient charge-transfer process and stronger molecular interactions within the imprinted polymer layer. The higher proportion of 4-vinylpyridine in this formulation appears to facilitate more stable and energetically favorable binding with the collagen template, resulting in improved site fidelity and greater accessibility during the rebinding step.

Condition 3, where the proportion of AAM is increased relative to VP, the cyclic voltammetry curves reveal a noticeable enhancement in current response compared to Condition 1. The anodic and cathodic features become more defined as collagen concentration increases, reflecting stronger hydrogen-bonding interactions between the amide-rich polymer matrix and the collagen template. The relative peak shifts and current intensities indicate that the higher AAM content promotes a microenvironment resembling a hydrated hydrogel, which is favorable for protein accessibility and rebinding. However, the improvement is not entirely uniform across the concentration range. At lower concentrations, the CV curves display greater variability, and the peak symmetry becomes less consistent as indicates in Figure 5c. This suggests that while AAM enhances chemical affinity, an excessive proportion may lead to a denser polymer network that restricts efficient charge transfer and slows analyte diffusion toward the electrode surface. As a result, although the high-concentration responses approach 30% current change slightly higher than Condition 1, the reproducibility and sensitivity at low analyte levels remain limited.

Overall, Condition 3 demonstrates better binding strength than Condition 1 but lacks the balanced network structure required for stable, dose-dependent electrochemical performance. This supports the conclusion that an optimal ratio of AAM and VP instead of AAM-dominant formulations is necessary to achieve selective and efficient type II collagen detection.

**Figure 6:**
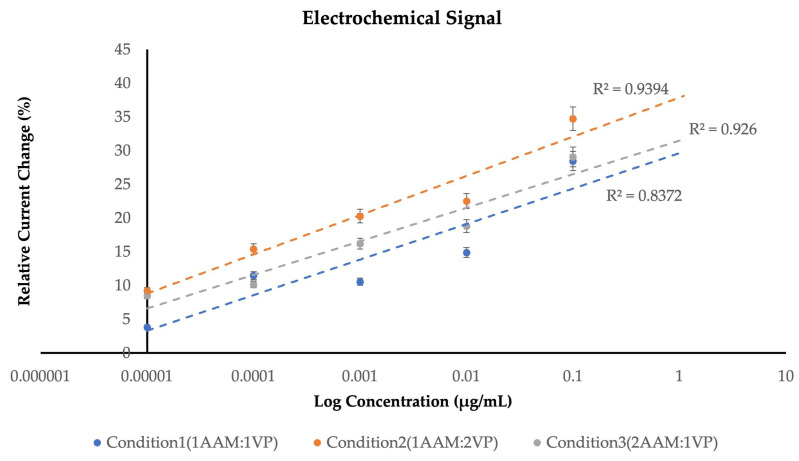
Comparison of percentage relative current change as a function of collagen concentration under three different sensing conditions.

Figure 6 showing the comparative analysis of the electrochemical responses revealed clear differences among the three polymer conditions across the tested collagen concentrations. Condition 1 (1AAM:1VP) generated the weakest signal progression (R^2^ = 0.8372), characterized by a shallow slope and only moderate linearity, indicating limited responsiveness and a lower affinity toward collagen rebinding. Condition 3 (2AAM:1VP) demonstrated slightly improved performance (R^2^ = 0.9260), with a more noticeable rise in relative current change; however, the overall trend remained less efficient, suggesting that the elevated AAM content may restrict optimal charge transfer within the polymer network. In contrast, Condition 2 (1AAM:2VP) exhibited the most pronounced and consistent enhancement in electrochemical output. The signal increased sharply with concentration, yielding the steepest calibration slope and the strongest correlation coefficient (R^2^ = 0.9394). This improvement reflects more effective molecular recognition and electron-transfer kinetics, likely driven by the higher proportion of VP, which enhances intermolecular interactions and facilitates clearer differentiation in current response across the concentration range.

The analytical performance of the sensor was further assessed through calculation of the limit of detection (LOD), using the standard approach of 3σ divided by the slope of the calibration curve, as follows

(1) $$\mathrm{LOD}=\frac{3\sigma }{Slope}\;,$$

Based on the response obtained across the tested concentration range (0.01–100 µg/mL), the LOD was determined to be approximately 0.065 µg/mL. This low detection threshold demonstrates the sensor’s strong capability to resolve subtle current changes at minimal collagen levels, confirming its suitability for early-stage analyte monitoring where concentrations are typically limited.

In addition to the detection limit, the limit of quantification (LOQ) was determined to further evaluate the analytical capability of the sensor. Using the 10σ calibration approach. Using the equation

(2) $$\mathrm{LOQ}=\frac{10\sigma }{Slope}\;,$$

The LOQ was estimated to be approximately 0.21 µg/mL, indicating the concentration at which the sensor begins to deliver consistently precise and reproducible measurements. This quantification threshold, together with the low LOD, highlights the sensor’s capacity to operate effectively within the biologically relevant range for collagen degradation analysis.

### 3.2. Characterization

Scanning Electron Microscopy (SEM) was chosen to characterize the morphology of the molecularly imprinted polymer and to verify that the imprinting process successfully generated cavities complementary to type II collagen on the electrode surface. After polymerization and template removal, the modified electrodes were examined under SEM to observe changes in surface topology, compare the imprinted layer with non-imprinted controls, and assess the presence, uniformity, and distribution of the formed recognition sites. Particular attention was given to identifying porous features that reflect the spatial arrangement left by the extracted collagen molecules. This structural evaluation provides essential confirmation that the polymer network retained the expected cavity architecture, which is critical for selective rebinding of type II collagen during electrochemical sensing.

**Figure 7:**
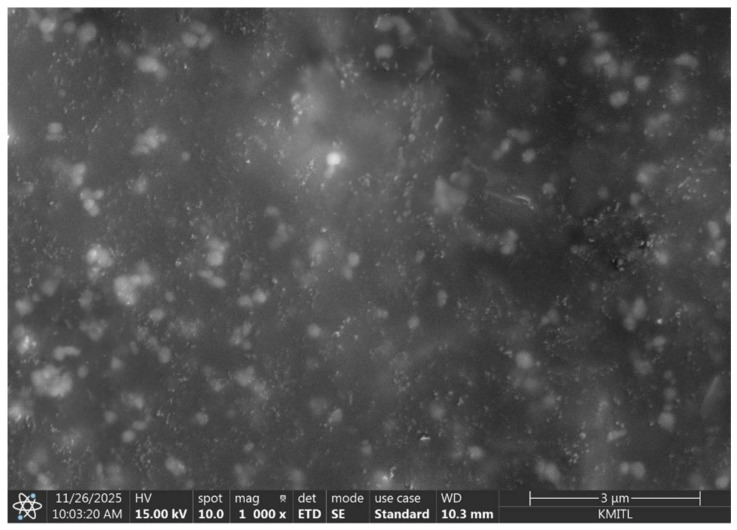
The SEM image on carbon electrode surface before polymerization.

Figure 7 presents scanning electron microscopy (SEM) images of the corresponding non-imprinted polymer (NIP) control. The NIP surface exhibits a relatively smooth and homogeneous polymer layer with the absence of well-defined cavities, reflecting polymer formation in the absence of the collagen type II template.

**Figure 8:**
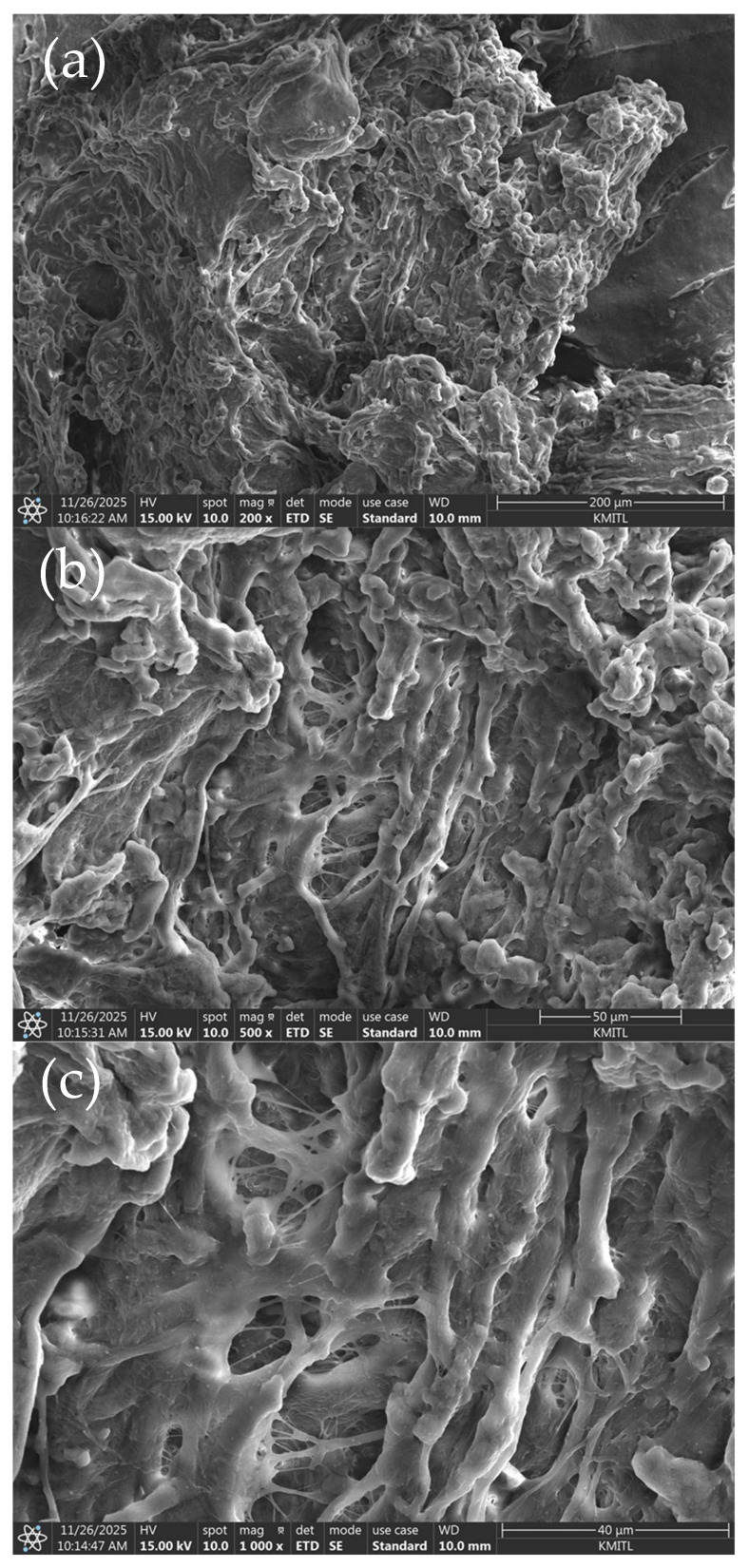
The SEM image on carbon electrode surface after polymerization (**a**) 200× magnifying power, (**b**) 500× magnifying power, and (**c**) 1000× magnifying power.

In contrast, Figure 8 the SEM image shows morphology on electrode surface at each resolution. At 200×, the image shows an overall heterogeneous and rough surface, indicating that the polymerization process generated a non-uniform topology consistent with the presence of imprinted structures across the electrode. Moving to 500×, more defined porous features become visible, revealing irregular depressions and open channels distributed throughout the polymer matrix. These features suggest the partial collapse and restructuring of the polymer surrounding the template during imprint formation. At the highest magnification, 1000×, the image clearly displays a highly interconnected network of voids and fibrous structures, providing strong visual evidence of the nanoscale cavities left by the extracted type II collagen molecules. Together, these images confirm that the imprinting procedure successfully created a cavity-rich surface architecture capable of selectively rebinding the target collagen during sensing.

### 3.3. Selectivity Test

The triple-helix structures of types I, II, and III collagen share a common backbone composed of repeating glycine–proline–hydroxyproline motifs, giving them closely related molecular architectures. Despite this overall similarity, each type carries subtle variations in amino-acid sequence and helix flexibility that influence its biological function and molecular interactions. Type I collagen forms a tightly packed and mechanically robust helix, characteristic of tissues such as tendon, bone, and skin where high tensile strength is required. Type II collagen exhibits a slightly more compliant helical arrangement, enabling the resilience and compressive resistance essential for cartilage. Type III collagen, by contrast, adopts a more delicate and elastic triple helix, aligning with its role in vascular and soft connective tissues. Because these fibrillar collagens share structural motifs yet differ in fine molecular details, their electrochemical behavior at the sensor interface cannot be assumed to be identical. Even minor differences in surface charge distribution, flexibility, and residue orientation can influence how each type reorganizes the microenvironment around the imprinted cavities. Therefore, performing a specific binding test across types I, II, and III collagen is essential to verify that the MIP sensor preferentially recognizes type II rather than responding nonspecifically to structurally related collagens. This comparison ensures that the observed signal shifts originate from true template–polymer affinity, thereby confirming the selectivity and diagnostic relevance of the sensor.

**Figure 9:**
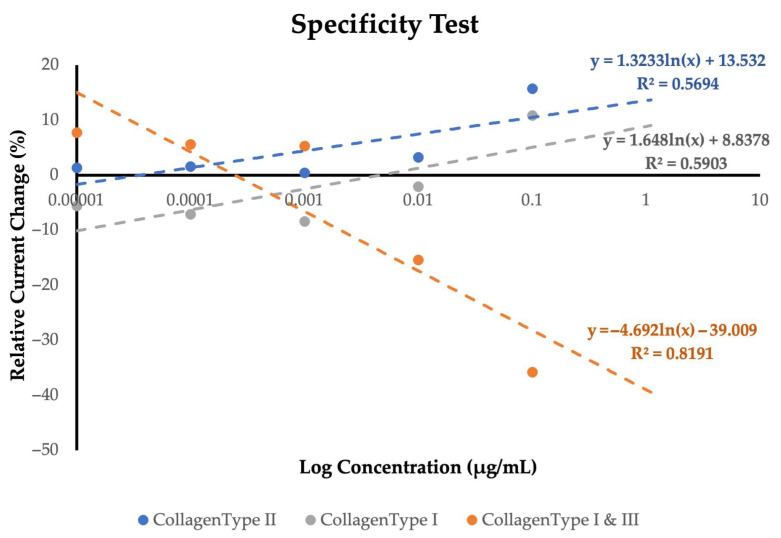
Shows the comparison of percentage in relative current change between 3 types of collagens analyte.

Figure 9 illustrates the specificity assessment of the MIP-based sensor by comparing the electrochemical responses to collagen type II, collagen type I, and the mixed type I & III sample. The curve corresponding to collagen type II exhibits a clear upward trend, with relative current change increasing progressively across the tested concentrations. Although the correlation coefficient is moderate, the overall pattern reflects a consistent and concentration-dependent interaction, supporting its role as the true target molecule. In contrast, collagen type I produces only minimal variations in signal intensity, resulting in a shallow slope and a notably weaker correlation. This limited response indicates poor affinity toward the imprinted cavities, reinforcing the selectivity of the polymer toward type II. A markedly different behavior is observed for the combined type I & III sample. The sharp negative slope and substantial signal reduction with increasing concentration do not follow the profile of a genuine binding event. Instead, this pattern suggests non-specific processes or competitive surface effects rather than meaningful molecular recognition. Collectively, these outcomes confirm that the sensor responds preferentially and most reliably to collagen type II under the tested conditions.

## 4. Discussion

The analytical performance of the fabricated sensor was examined using type II collagen as the target analyte across a broad concentration range (0.01–100 µg/mL). Based on the calibration data, compared with established techniques such as ELISA and UHPLC-MS/MS reported in the literature [14], which typically exhibit detection limits around 0.5 µg/mL for type II collagen, the proposed MIP-based electrochemical sensor achieved a markedly lower limit of detection (0.065 µg/mL). This low detection threshold highlights the sensor’s capability to register subtle variations in collagen abundance, which is essential for early detection of cartilage-related degeneration and potential suitability for point-of-care applications.

Among all polymer formulations evaluated, the 1AAM:2VP composition consistently outperformed the others. This ratio generated the steepest calibration gradient and produced the most coherent signal progression, indicating efficient molecular recognition and favorable charge-transfer kinetics at the electrode interface. The strong linearity (R^2^ = 0.9394) further confirms that this polymer network provides a stable and predictable response over several orders of magnitude in concentration. This behavior suggests that the increased proportion of VP promotes more effective binding interactions with collagen type II, enhancing both structural compatibility and electron-transfer behavior within the imprinted cavities.

Selectivity assessments further support the suitability of the optimized formulation for type II collagen sensing. Small changes in current were observed for collagen type I, and the mixed type I & III sample produced an inconsistent, declining signal profile rather than a characteristic binding-induced response. These findings demonstrate that the imprinted polymer matrix discriminates well between structurally similar collagen species, reflecting successful imprint formation and target-specific cavity retention after template removal.

Overall, the electrochemical and selectivity outcomes indicate that the 1AAM:2VP formulation provides a robust foundation for selective and sensitive detection of collagen type II. While the present study focused on electrochemical characterization, photocharacteristic evaluation will be incorporated in future work, pending availability of specialized equipment and additional development time.

## 5. Conclusions

In this research, a molecularly imprinted polymer–based electrochemical sensor was successfully developed for the selective detection of collagen type II. The optimized formulation, incorporating a 1AAM:2VP monomer ratio, exhibited the highest electrochemical responsiveness, superior linearity, and enhanced binding efficiency across the tested concentration range. The sensor achieved an LOD of approximately 0.065 µg/mL, demonstrating strong analytical sensitivity suitable for early detection of collagen degradation. Selectivity studies further confirmed that the imprinted layer preferentially recognized collagen type II over structurally similar collagen types I and I&III, validating the specificity of the molecular imprinting strategy. These findings highlight the potential of this platform for future use in cartilage health monitoring and degenerative joint disease screening.

## Figures and Tables

**Table 1:** Cyclic voltammetry signal characteristics of MIP sensors fabricated with varying AAM: VP ratios.

Functional Monomers Ratio	Sample Test Concentrations (µg/mL)	Current Signal Height (µA)	∆I (µA)	Relative Current Change (%)
Condition 1 1AAM:1VP	Blank	30.87	0	0
	0.01	29.69	1.18	3.82
	0.1	27.33	3.54	11.46
	1	27.62	3.26	10.54
	10	26.28	4.59	14.87
	100	22.10	8.78	28.43
Condition 2 1AAM:2VP	Blank	30.01	0	0
	0.01	27.24	2.76	9.20
	0.1	25.38	4.62	15.39
	1	23.91	6.08	20.28
	10	23.25	6.75	22.51
	100	19.58	10.41	34.72
Condition 3 2AAM:1VP	Blank	34.57	0	0
	0.01	24.52	2.94	8.50
	0.1	28.07	3.51	10.15
	1	28.97	5.60	16.19
	10	31.06	6.50	18.79
	100	31.63	10.05	29.06

## Data Availability

The raw data supporting the conclusions of this article will be made available by the authors on request.

## Abbreviations

The following abbreviations are used in this manuscript:

AAM	Acrylamide
AIBN	Azobisisobutyronitrile
CE	Counter Electrode
CV	Cyclic Voltammetry
DMSO	Dimethyl Sulfoxide
DJD	Degenerative Joint Diseases
EGDMA	Ethylene Glycol Dimethacrylate
ELISA	Enzyme-linked immunosorbent assays
GO	Graphene Oxide
LOD	Limit of Detection
LOQ	Limit of Quantity
MIP	Molecularly Imprinted Polymer
OA	Osteoarthritis
PCR	polymerase chain reaction
PVP	Polyvinylpyrrolidone
RA	Rheumatoid arthritis
RE	Reference Electrode
SEM	Scanning Electron Microscopy
SPE	Screen-Printed Electrode
TMJ	temporomandibular joint
UPLC-MS/MS	Ultraperformance liquid chromatography–tandem mass spectrometry
UV	Ultraviolet
VP	N-vinylpyrrolidone
WE	Working Electrode
